# Current status in whole genome sequencing and analysis of *Ipomoea* spp.

**DOI:** 10.1007/s00299-019-02464-4

**Published:** 2019-08-29

**Authors:** Sachiko Isobe, Kenta Shirasawa, Hideki Hirakawa

**Affiliations:** Kazusa DNA Research Institute, Kazusa-Kamatari 2-6-7, Kisarazu, Chiba Japan

**Keywords:** Sweet potato, *Ipomoea*, Whole genome, Sequencing

## Abstract

The recent advances of next-generation sequencing have made it possible to construct reference genome sequences in divergent species. However, de novo assembly at the chromosome level remains challenging in polyploid species, due to the existence of more than two pairs of homoeologous chromosomes in one nucleus. Cultivated sweet potato (*Ipomoea batatas* (L.) Lam) is a hexaploid species with 90 chromosomes (2*n* = 6*X* = 90). Although the origin of sweet potato is also still under discussion, diploid relative species, *I. trifida* and *I. triloba* have been considered as one of the most possible progenitors. In this manuscript, we review the recent results and activities of whole-genome sequencing in the genus Ipomoea series Batatas, *I. trifida, I. triloba* and sweet potato (*I. batatas*). Most of the results of genome assembly suggest that the genomes of sweet potato consist of two pairs and four pairs of subgenomes, i.e., B1B1B2B2B2B2. The results also revealed the relation between sweet potato and other *Ipomoea* species. Together with the development of bioinformatics approaches, the large-scale publicly available genome and transcript sequence resources and international genome sequencing streams are expected to promote the genome sequence dissection in sweet potato.

## Introduction

Genome and gene sequences have become essential information for a wide range of biological studies. A decade ago, high-quality reference sequences were available only for model or well-studied organisms. The recent advances of next-generation sequencing (NGS), however, have made it possible to construct reference genome sequences in divergent species, including plants. In plants, a total of 319 species have been registered as whole genome-sequenced species in the plaBiPD database (https://www.plabipd.de/, 2019 May). In addition to the number of sequenced species, the quality in de novo assembly of genomes has also been improved with advances in long reads sequencing technologies, such as the PacBio (Pacific Biosciences, Menlo Park, CA, USA) and Nanopore (Oxford Nanopore Technologies, Oxford, UK) sequencing platforms. Thus, the performance of reference-grade whole-genome sequencing has become common over the past 5 years.

Advances in the bioinformatics of genome assembly are another key factor supporting the spread of de novo whole-genome sequencing in various species. Algorithms for genome assembly were first developed for haploid or diploid species, with a few for polyploid species. Therefore, construction of reference genomes continues to be less advanced in polyploid species than in diploids. Polyploidy is often observed in plant species and contributes to human life through crops such as wheat (*Triticum aestivum*), potato (*Solanum tuberosum*), strawberry (*Fragaria* × *ananassa*), coffee (*Coffea arabica*), cotton (*Gossypium hirsutum*), and sweet potato (*Ipomoea batatas*). However, de novo assembly at the chromosome level remains challenging in polyploid species, due to the existence of more than two pairs of homoeologous chromosomes in one nucleus. By 2018, only a total of 16 polyploid species had chromosome-level reference genomes (Kyriakidou et al. 2018). This was achieved due to advances in NGS technologies and bioinformatics. In addition, reference sequences of diploid relatives have helped to resolve the complexity of polyploid genomes. Therefore, reference genome constructions in related diploid species were often performed prior to de novo assembly in polyploid genomes (Bertioli et al. 2016; D’Hont et al. 2012; Ling et al. 2013; Shulaev et al. 2011; Wang et al. 2012).

Cultivated sweet potato (*Ipomoea batatas* (L.) Lam) is a hexaploid species with 90 chromosomes (2*n* = 6*X* = 90), and the structure of the hexaploid genome has not yet been defined. It was first considered an allohexaploid species based on cytological studies (Jones 1967; Magoon et al. 1970; Sinha and Sharma 1992). Later, several studies suggested that the genome structure of sweet potato was autohexaploid, based on the inheritance behaviors of DNA markers in linkage analysis (Cervantes-Flores et al. 2008; Kriegner et al. 2003; Ukoskit and Thompson 1997; Zhao et al. 2013).

The origin of sweet potato is also still under discussion. The genus *Ipomoea* includes approximately 500–600 species, and sweet potato is the only species cultivated in the genus *Ipomoea* series *Batatas* (Austin 1988). Thirteen wild species are considered to be closely related to sweet potato (Austin 1988; Austin and Huáman 1996). Of these, *I. trifida* (H.B.K.) Don. has been considered the most likely progenitor (Nishiyama 1971; Shiotani and Kawase 1989). Nishiyama considered that sweet potato was derived from the hexaploid *I. trifida,* which was generated from the hybridization of the diploid *I. leucantha* Jacq. and tetraploid *I. littoralis* Blume. Shiotani and Kawase (1989) also hypothesized that the origin of sweet potato was hexaploid *I. trifida*, based on the development of an artificial hybridization of the diploid and tetraploid *I. trifida* accessions. Later, the hypothesis was strongly supported by Munoz-Rodrıguez et al. (2018) based on gene sequences comparison in genus *Ipomoea* and Wu et al. (2018) with whole-genome sequencing in sweet potato.

The genome structures of diploid and tetraploid accessions were assumed to be B1B1 and B2B2B2B2, respectively; therefore, that of sweet potato was considered to be B1B1B2B2B2B2, a mixture of allo and auto polyploidy. A similar genome structure (AABBBB) was also suggested by Reddy et al. (2007). However, they considered that three *Ipomoea* species were possible ancestors, i.e., diploid *I. triloba* (A genome), diploid *I. trifida* (B genome), and tetraploid *I. tabascana* (B genome).

It is expected that the whole genome sequence would reveal the features of the sweet potato genome, and the relation between sweet potato and other *Ipomoea* species. Construction of the reference genome sequence in sweet potato would also contribute to advances in genetic, genomic, and physiological studies of the species. In this manuscript, we review the recent results and activities of whole-genome sequencing in the genus *Ipomoea* series *Batatas*, including sweet potato.

## Genome sequencing in diploid relatives of sweet potato, *I. trifida* and *I. triloba*

The first de novo whole-genome sequencing in diploid relatives of sweet potato was reported by Hirakawa et al. (2015) in *I. trifida* (Table 1). Two lines, Mx23Hm and 0431-1, were sequenced using the Illumina HiSeq platform. Mx23Hm is a single descendant selfed line (S11) derived from Mx23-4, while 0431-1 is a highly heterozygous line. The two lines were selected for sequencing because Mx23-4, the ancestral line of Mx23Hm, and 0431-1 were used as parental lines for AFLP linkage map construction (Nakayama et al. 2010). Paired-end (PE) reads were assembled by SOAPdenovo2 r223 (Li et al. 2010), and scaffolding was done with mate pair (MP) reads by SSPACE2.0 (Boetzer et al. 2011). Mx23-4 generated 77,400 scaffolds with 513.0 Mb length, while 0431-1 had 181,194 that were 712.2 Mb in length. The estimated genome sizes in Mx23Hm and 0431-1 based on k-mer frequency analysis were 515.8 Mb and 539.9 Mb, respectively.

**Table 1:** Assembled nucleic whole genome sequences in genus *Ipomoea* series Batatas

Species	Polyploidy	Estimated genome size (Mb)	Heterozygosity	Sequenced line name	Sequence reads	Scaffolds			Pseudo-molecule length (Mb)	Number of predicted genes	References
						Number of sequences	Total length (bp)	N50 length (bp)			
*I. trifida*	Diploid	515.8	Few	Mx23Hm	Illumina (PE, MP)	77,400	512,990,885	42,586	–	62,407	Hirakawa et al. (2015)
*I. trifida*	Diploid	539.9	High	0431-1	Illumina (PE, MP)	181,194	712,155,587	36,283	–	109,449	Hirakawa et al. (2015)
*I. trifida*	Diploid	526.4	High	NCNSP0306	Illumina (PE, MP), PacBio, BioNano (Irys)	30,394	461,997,559	1,237,020	373.4	44,158	Wu et al. (2018)
*I. triloba*	Diploid	495.9	Few	NCNSP0323	Illumina (PE, MP), PacBio, BioNano (Irys)	4008	457,835,428	6,861,300	443.3	47,008	Wu et al. (2018)
*I. trifida*	Diploid	476.4	High	Y22	Illumina (PE, MP, SLR), PacBio	5264	460,931,543	607,924	400.4	30,227	Li et al. (2019)
Sweet potato *(I. batatas*)^a^	Hexaploid		High	Taizhong6	Illumina (PE, MP), Roche 454 (SE)	35,919	836,316,092	200,728	633.4	78,781	Yang et al. (2017)

^a^Scaffolds statistics represent haplotype-improved assembly

The total length in the assembled genome of 0431-1 was longer than the estimated genome size, probably due to probable separated haploid-level assemblies in highly heterozygous regions. The two assembled genomes were compared, and the assembled sequences were classified into core candidates, conserved between the two lines, and line-specific sequences. The total lengths of the core candidate sequences were 240 Mb (Mx23Hm) and 353 Mb (0431-1). The numbers of predicted genes in Mx23Hm and 0431-1 were 62,407 and 109,449, respectively. Although the assembled sequences were not chromosome level, they contributed to the advances in sweet potato studies as the first reference genomes (Shirasawa et al. 2017; Si et al. 2016; Zhang et al. 2017).

The first chromosome-level references in *I. trifida* and *I. triloba* were constructed by Wu et al. (2018) using a heterozygous line of *I. trifida*, NCNSP0306, and a highly homozygous line of *I. triloba*, NCNSP0323 (Table 1). Illumina PE and MP reads were obtained for both species, and assembly was performed by Platanus v1.2.1 (*I. trifida;* Kajitani et al. 2014) and SOAPdenovo2 (*I. triloba*). PacBio reads were used for gap filling, and BioNano genome maps were used to improve the assembly. In *I. trifida*, 30,394 scaffolds were generated with a total length of 462.0 Mb, whereas in *I. triloba* there were 4008 with a total length of 457.8 Mb. The high homozygosity of the sequenced line of *I. triloba* resulted in the longer length of the assembled scaffolds. A genotyping by sequencing (GBS)-based linkage map was constructed based on an F_1_*I. trifida* mapping population for pseudomolecule construction. Each group of 15 pseudomolecules were generated in *I. trifida* and *I. triloba* by aligning the scaffolds on the linkage map. The total length of pseudomolecules in *I. trifida* and *I. triloba* was 373.4 Mb (80.8% of the assembly) and 443.3 Mb (96.8% of the assembly), respectively. The numbers of predicted protein-encoding genes in *I. trifida* and *I. triloba* were 32,301 and 31,423, respectively.

Illumina transcript read assembly was also performed in the sweet potato cultivar Beauregard, and the 43,296 generated protein sequences were used for comparative analysis with predicted proteomes from *I. trifida* and *I. triloba*. A total of 1680 Batatas complex specific protein clusters were identified in a comparison with other seven species *I. nil* (Japanese morning glory), tomato, potato, grapevine, Arabidopsis, rice, and *Amborea trichopoda* (basal angiosperm). The existence of whole-genome triplication in the genus *Ipomoea* was also suggested by comparisons with the grape genome. The utility of assembled genomes was also investigated by mapping 10× Genomics Chromium reads of sweet potato variety Tanzania onto the *I. trifida* and *I. triloba* genome assemblies. Although 390,303 regions in Tanzania were homologous to both assemblies, the existence of *I. trifida*- or *I. triloba*-specific regions suggested that the sweet potato genome contains sequences that are uniquely shared with the two species. In addition, Wu et al. (2018) accounted that contribution of *I. trifida*-like progenitor was approximately twice that of *I. triloba*-like progenitor. The result proves the hypothesis that the sweet potato genome structure is B1B1B2B2B2B2. The first established pseudomolecules in the two *Ipomoea* species has contributed revealing insight into the sweet potato genome.

Another assembled genome sequence at the chromosome level was generated in an *I. trifida* variety, Y22, which forms a storage root (SR) (Li et al. 2019, Table 1). Illumina PE and MP reads were assembled by Platanus, and Moleculo synthetic long reads (SLRs) were used for gap filling. PacBio reads were further used to extend sequence continuity, and the sequence redundancy caused by heterozygosity was excluded using HaploMerger (Huang et al. 2012). As a result, 5264 scaffolds were generated with a total length of 460.9 Mb. A GBS-based linkage map derived from an F_1_ mapping population (Y25 × Y22) was constructed, and the 15 pseudomolecules were generated by aligning the scaffolds onto the linkage map. The total length of the pseudomolecules was 440.4 Mb, covering 86.9% of the assembled scaffolds. A total of 30,227 genes were predicted on the assembled scaffolds with the support of transcript reads from seven tissues in *I. trifida*—namely, leaf, flower, stigma, pollen, stem, root and seed.

The divergence time of *I. trifida* and *I. nil* was estimated as 6.4 Mya, based on 1930 single-copy genes. This was earlier than the estimation of 3.6 Mya by Wu et al. (2018). The key genes in starch accumulation were further investigated based on RNA-Seq analysis in four different stages of root development, QTL mapping, and comparisons of gene families with other species, *I. nil*, tomato, *Coffea canephora* and Arabidopsis. Based on the obtained results, it was predicted that the beta-amylase gene family might be related to SR-formation, with *BMY11* being the most responsible gene. Li et al. (2019) also deduced that the function of *BMY11* was to split smaller starch granules in cells to synthesize larger starch granules, based on the expression pattern of BMY11 in *I. trifida* (Y22) and sweet potato (Xushu 18), and anatomical observation at different stages of SR development. Identification of a candidate gene relating SR development in *I. trifida* in this study suggested that sweet potato obtained ability of starch accumulation in root via mutation of gene expression in the *I. trifida*-like progenitor. The study also indicated the importance of existence of high-quality *I. trifida* reference genome to accelerate gene function analysis in sweet potato.

## Genome sequencing in sweet potato

Yan et al. (2015) constructed a complete chloroplast (cp) genome in sweet potato with Illumina PE and MP reads of a Chinese cultivar, Xushu18. The sequences were assembled using Edena v2.1.1 (Hernandez et al. 2008), SOAPdenovo2 r240 and Velvet v1.0.12 (Zerbino and Birney 2008), and were combined by CD-HIT-EST and CAP3 (Huang and Madan 1999). The organellar sequences were isolated from the nuclear genome based on mapped read depth and number of copies. Then, cp genome sequences were identified by a BLAST search against *I. trifida* cp DNAs reported by Eserman et al. (2014). The isolated sequences were assembled into a circular molecule of 161,303 bp. A total of 145 genes were predicted on the genome, including 72 single- and 11 double-copy protein-encoding genes. Gene-flow and gene-gain-and-loss events were detected by comparing the chloroplast sequences of 33 species. Moreover, RNA-editing events and differential expressions of the chloroplast functional genes were identified by comparing sweet potato transcript sequences.

Si et al. (2016) obtained genome-wide BAC-end sequences (BESs) using the Sanger method and investigated the features of the sweet potato genome. A total of 8310 BAC clones randomly selected from the 240,384 clones were sequenced at both ends, generating high quality 111,542 BESs with a total length of 7595,261 bp after trimming vector and low-quality sequences from 16,620 raw data. Known and unique sweet potato repetitive sequences accounted for 12.2% and 18.3% of the BESs, respectively. Based on the analysis of BESs, 10% of the sweet potato genome was estimated to consist of coding regions. The density of simple sequence repeats (SSRs) was estimated at one SSR per 1.93 Kb. It was a first report of genome sequencing in sweet potato and provided a platform for genetic and genomic studies such as DNA marker development and gene cloning.

Yang et al. (2017) reported the first whole-genome de novo assembly in sweet potato (Table 1). A carotenoid-rich cultivar, Taizhong6, was used for genome sequencing, and libraries were constructed for Illumina PE, MP and Roche 454 single-end (SE) reads. The researchers developed a unique pipeline to constructed haplotype-resolved genome sequences. Preliminary assembly was performed first using IDBA-UD (Peng et al. 2012), Newbler 3.0 and Platanus, and a total of 57,051 sequences were generated with 831.9 Mb length. Then, variants among the homoeologous chromosomes were identified by mapping all Illumina reads. The 14,342,083 identified variants were used as seeds for haplotype phasing, and ~ 30% of the genome was phased into six haplotypes. Phased haplotypes were merged by overlapped sequences or PE reads. All the Illumina reads were mapped again onto the merged haplotype sequences and perfectly matched PE reads were used for haplotype connection. In this way, a haplotype-improved assembly was generated.

The haplotype-improved assembly included a total of 35,919 scaffolds. The total and N50 lengths of the scaffolds were 836.3 Mb and 200.7 Kb, respectively. Fifteen pseudomolecules were constructed by anchoring 7470 of the 35,919 scaffolds to the *I. nil* genome (Hoshino et al. 2016). The total length of the 15 pseudomolecules was 633.4 Mb or 75.7% of the haplotype-improved assembly. By mapping transcript sequences of sweet potato generated in different tissues, 78,781 gene models were extracted on the 15 pseudomolecules. Haplotype-resolved regions were further identified based on the 15 pseudomolecules. A total of 644,810 regions were successfully phased and variants among homoeologous chromosomes were investigated. Phylogenetic analysis was also performed for the haplotype-resolved sequences, and the branching patterns of two haplotypes versus four haplotypes were identified on a UPGMA tree. The results suggested that the genome structure of sweet potato was B1B1B2B2B2B2. A dominant division of two versus two was also identified in the four-haplotype subgroup, suggesting the possibility of two whole-genome duplication (WGD) in sweet potato. Based on the mutation rate, it was predicted that the first and second WGD occurred 0.8 MYA and 0.5 MYA ago. Although miss-assembly in the constructed genome sequences was pointed out in later by Wu et al. (2018), it was a first report of whole-genome de novo assembly and contributed to reveal genome structured in sweet potato.

## Databases

Currently, four databases (DBs) are available for genome sequences of sweet potato and its related species.

1. Sweet potato Genomic Resource (http://sweetpotato.plantbiology.msu.edu/)

This DB provides *I. trifida* and *I. triloba* genome sequences published by Wu et al. (2018). It is hosted by Michigan State University with the support of the GT4SP Improvement Project and the Bill and Melinda Gates Foundation. The DB has a genome browser (JBrowse), BLAT search, Annotation search and e-PCR tool. It is the most active DB in sweet potato genomic resources and data was renewed several times.

2. Ipomoea Genome Hub (https://ipomoea-genome.org/)

This DB provides sweet potato genome sequences published by Yang et al. (2017). It is hosted by the Shanghai Chenshan Botanical Garden, Max Planck Society, and Chinese Academy of Sciences. The DB has genome browsers (JBrowse and GBrowse), and a BLAST search. Because the Shanghai Chenshan Botanical Garden hosts the DB, the website is also connected to an image-based DB (the Ipomoea Atlas).

3. Ipomoea Batatas Genome Browser (http://public-genomes-ngs.molgen.mpg.de/SweetPotato/)

This DB stores sweet potato genome sequences published by Yang et al. (2017). It is hosted by MPI Molecular Genetics. The DB has a genome browser and BLAST search.

4. Sweet potato GARDEN (http://sweetpotato-garden.kazusa.or.jp/)

This DB provides *I. trifida* genome sequences published by Hirakawa et al. (2015) and is hosted by the Kazusa DNA Research Institute. The DB allows a BLAST search against genome, CDS and protein sequences. KEGG maps and a genetic map are also available.

5. *Ipomoea nil* (http://viewer.shigen.info/asagao/)

This DB provides *I. nil* genome sequences published by Hoshino et al. (2016). It is hosted by the Morning glory genome Consortium. The DB has genome browsers (JBrowse), and BLAST and BLAT searches.

## Future perspectives

Recent progress in whole-genome sequences in sweet potato and its wild diploids has contributed to our understanding of the features of genome structures and evolutionary events. It is particularly worth noting that most of the results of genome assembly suggest that the genomes of sweet potato consist of two pairs and four pairs of subgenomes, i.e., B1B1B2B2B2B2. This fact also suggests that sweet potato remains a tough species for genetic and genomic analysis, because the discussion of statistics and bioinformatic approaches in mixtures of allo- and auto-polyploidy genomes is still in the early stages. To advance the exploration of this topic and for downstream use, it would be necessary to further enhance the quality of the reference genome of sweet potato. For example, Wu et al. (2018) found significant numbers of miss-assemblies in the sweet potato haplotype-resolved assembly constructed by Yang et al. (2017). Therefore, the GT4SP program, which develops next-generation breeder tools for African sweet potato breeders, had communicated with the authors of Yang et al. (2017) for evaluation of the sweet potato genome to use downstream analysis.

One international genome-sequencing project is ongoing by the Trilateral Research Association of Sweet potato (TRAS) genome-sequencing consortium (Yoon et al. 2015). The consortium was launched in 2012, and consists of six organizations, the Jiangsu Xuzhou Sweet Potato Research Center (China), China Agricultural University (China), Rural Development Administration (Korea), Korea Research Institute of Bioscience and Biotechnology (Korea), National Agriculture and Food Research Organization (Japan), and Kazusa DNA Research Institute (Japan). Haplotype-based assembly has been attempted with PacBio and Illumina reads. The NGS technologies have continually advanced, with improvements to both the quality and quantity of reads. Although capturing the sweet potato genome is still difficult at present, it is expected that we will find better solutions step by step, as has been the case in past studies.

Together with the development of bioinformatics approaches, the large-scale publicly available genome and transcript sequence resources and international genome sequencing streams are expected to promote the genome sequence dissection in sweet potato.

### Author contribution statement

SI wrote the whole manuscript as a corresponding author. KS and HH investigated and modified the manuscript.
